# Neutralizing antibody against GDF15 for treatment of cancer-associated cachexia

**DOI:** 10.1371/journal.pone.0309394

**Published:** 2024-08-22

**Authors:** Junyi Xiong, Guojin Wu, Jinying Ning, Junlin Yan, Jian Yang, Jinsen Kang

**Affiliations:** 1 College of Pharmacy, Xinjiang Medical University, Urumqi, Xinjiang, China; 2 KYINNO Biotechnology (Beijing) Co., Ltd., Beijing, China; 3 Engineering Research Center of Xinjiang and Central Asian Medicine Resources, Ministry of Education, Urumqi, Xinjiang, China; University of Cambridge, UNITED KINGDOM OF GREAT BRITAIN AND NORTHERN IRELAND

## Abstract

GDF15 (growth differentiation factor 15), also known as macrophage inhibitory cytokine 1 (MIC-1), is a circulating protein involved in the regulation of energy balance and weight control. Elevated levels of GDF15 have been associated with cachexia and reduced survival rates in cancer patients. Through the activation of the GFRAL (GDNF-family receptor α-like)-RET (Rearranged during Transfection) signaling pathway, GDF15 can induce weight loss, making it a potential target for treating cachexia. Currently, there are no approved antibody drugs specifically targeting GDF15 for cancer cachexia treatment. However, efforts have been made to develop antibody-based therapeutics against this emerging target. In this study, we generated a monoclonal antibody KY-NAb-GDF15 against GDF15 that effectively blocks downstream signaling mediated by GFRAL upon stimulation by GDF15. This antibody demonstrates robust neutralizing activity and exhibits high binding specificity. Importantly, our findings indicate that this antibody holds promise in alleviating cancer-induced cachexia and mitigating chemotherapy-induced weight loss, thereby offering significant therapeutic potential for managing cancer cachexia.

## Introduction

Cancer cachexia is a multifaceted syndrome characterized by involuntary weight loss, including depletion of skeletal muscle mass. Over time, it progresses to functional impairment and disrupts the homeostatic control of energy and protein balance [1–3]. Impaired muscle function can lead to reduced physical performance in cancer cachexia patients, resulting in compromised clinical treatment outcomes and diminished quality of life [4, 5]. Cachexia represents one of the major complication and cause of mortality among cancer patients [6, 7], yet there are currently no standardized treatment approaches or effective drugs specifically targeting cachexia. In recent years, multimodal therapy has been recommended as an adjunctive intervention for cancer cachexia [5, 8, 9]. Therefore, the development of efficacious drugs targeting cancer cachexia holds paramount importance in improving patients’ physical condition and enhancing treatment outcomes.

Growth differentiation factor 15 (GDF15), also known as macrophage inhibitory cytokine-1 (MIC-1), was discovered by Bootcov et al. in 1997. It functions as an autocrine regulatory molecule in macrophages and has been shown to regulate energy homeostasis under various pathological conditions, including cancer [10–13]. GDF15 has the potential to serve as a biomarker for cancer-related weight loss and a therapeutic target [14–16]. Research has found that GDF15 is a major causative factor of chemotherapy-induced cachexia [17, 18]. GDF15 regulates glial cell line-derived neurotrophic factor receptor alpha-like (GFRAL) activation, which is essential for its appetite-suppressing effects [19–22]. The cachexia-inducing effect of GDF15 is mediated through the formation of a ternary complex with GFRAL and its co-receptor RET [19–23]. In cancer patients, circulating levels of GDF15 are significantly elevated compared to those in healthy individuals [10, 24–26], and this elevation is associated with weight loss, poor prognosis, and decreased survival rates [27, 28]. Recent studies have also demonstrated that neutralizing GDF15 can improve anorexia and weight loss in animal models, alleviate side effects caused by chemotherapy, enhance the quality of life and survival rates in cancer patients [17, 28, 29].

The development and application of monoclonal antibodies targeting GDF15 for targeted cancer cachexia therapy represent a potentially effective approach to enhance cancer survival rates and treatment outcomes. NGM Biopharmaceuticals has successfully developed NGM120, a novel antagonistic antibody that binds to GFRAL and inhibits the signaling of GDF15, demonstrating promising potential in cancer treatment. Additionally, Rowena Suriben et al. [30] have reported on the therapeutic efficacy of an antagonistic monoclonal antibody 3P10 targeting GFRAL. Currently, there are no approved antibody drugs specifically targeting GDF15 for the treatment of cancer cachexia; however, as an emerging target, several pharmaceutical companies have developed corresponding antibody drugs to address this condition. These antibody drugs are still in the research and development stage, including Ponsegromab (Pfizer) [14], CTL-002 (CatalYm), AV-380 (AVEO), etc.

In this study, we utilized hybridoma technology to create KY-NAb-GDF15, a highly potent, specific, and high-affinity anti-GDF15 monoclonal antibody. The successful development of KY-NAb-GDF15 plays a pivotal role in advancing therapies for cancer cachexia and facilitating the progress of corresponding antibody-based treatments.

## Materials and methods

### Materials

The SP2/0, LS513, and HT1080 cell lines of multiple myeloma were acquired from the American Type Culture Collection (ATCC) and stored in our laboratory. Human GDF-15 / MIC-1 Protein, His Tag was procured from ACRO Biosystems. HRP-labeled goat anti-mouse IgG (Anti-Mouse IgG Fc specific-goat), HRP-labeled goat anti-human IgG (Anti-Human IgG Fc specific-goat), PE Goat anti-Mouse IgG, PE Goat anti-Human IgG Fc were obtained from SIGMA. Ponsegnomab, GFRAL-His, hGFRAL-hFc-Avitag, hGDF15-mFc(N), AV380-hIgG1-GDF15, Recombinant Human GDF-1 Protein, Recombinant Human GDF-3 Protein, Recombinant Human GDF-9 Protein, and Human IgG1, κ Isotype Control were sourced from KYINNO Biotechnology Co., Ltd. Tween20, BSA, TMB, 1×PBS (0.01 M pH 7.2–7.4), and ELISA stop solution were purchased from Solarbio Co., Ltd. BALB/c mice, C57BL/6 mice (females), B-hGDF15 mice (females), and female severe combined immunodeficiency (SCID) mice were obtained from HFK Bio Co., Ltd. All the mice were housed in specific pathogen-free facilities, and all animal experiments were approved by the Animal Ethics Committee of E-Town Biomedical Park in Beijing, China. All methods were conducted according to the Guide for the Care and Use of Laboratory Animals and relevant guidelines and regulations and reported following the Animal Research: Reporting of *In Vivo* Experiments (ARRIVE) guidelines for enhanced transparency.

### Monoclonal antibody preparation

Fifteen female BALB/c mice, aged 6 weeks, were randomly selected and divided into three groups for immunization via intramuscular, intraperitoneal, and subcutaneous injections. Each immunization comprised 10 μg of GDF15 protein mixed with an equal amount of aqueous adjuvant. Two weeks after the second injection, blood samples were collected from the mice’s eyeballs for titer detection using an indirect enzyme-linked immunosorbent assay (ELISA). The serum titers of the immunized mice were considered positive when OD450 nm > 2 compared to negative serum (N). Mice exhibiting higher titers received a subsequent injection of 20 μg GDF15 protein via intraperitoneal, intramuscular, or subcutaneous routes. Three days later, spleen cells from the immunized BALB/c mice were fused with SP2/0 myeloma cells through electrofusion. Positive hybridoma cells were screened using HAT medium through ELISA and further subcloning methods resulted in the selection of one stable hybridoma cell line secreting anti-GDF15 antibodies. The secreted anti-GDF15 antibody was designated as KY-NAb-GDF15 for subsequent experimentation.

### Binding affinity measurement

The Human GDF15-His protein was used as the coated antigen, while the KY-NAb-GDF15 antibody was employed as the test antibody with a series of dilutions. Subsequently, incubation with HRP-labeled Anti-Human IgG (Fc specific)-goat (1:10,000) was conducted. The OD450 values of each well were quantified using an enzyme immunoassay reader at a specific wavelength of 450 nm. The Human GDF15-His protein was employed as an antigen at a maximum working concentration of 200 nM and subsequently diluted. The detection probe underwent pre-treatment by immersion in the prepared buffer solution for a duration of 10 minutes. Finally, the Molecular Interaction Analyzer (Fortebio, OCTETRED96E) was utilized for detection. The HEK293 SRE-luc2-cRET-GFRAL reporter cell line is engineered to monitor the activity of the GDF15-driven interaction of RET with GFRAL on the cell surface. It contains a stable integration of Luciferase, a firefly luciferase gene, into HEK293 cells under the control of SRE response elements.

### Blocking function verification

The GFRAL-His protein serves as the coated antigen, while the hGDF15-mFC(N) protein acts as the blocking protein. The test antibody KY-NAb-GDF15 is diluted in gradients and incubated with HRP-labeled Anti-Human IgG (Fc specific)-goat (1:10,000). The OD450 values of each well are measured using a microplate reader at a single wavelength of 450 nm. For cellular level blocking experiments, HEK293 SER-Luc2-cRET-GFRAL cells constructed above are utilized. Initially diluted to 1 μg/mL, the antibody KY-NAb-GDF15 is co-incubated with 5 ng/mL GDF15 protein for 15 minutes before being added to the pre-seeded plate containing HEK293 SER-Luc2-cRET-GFRAL cells. A control group with only GDF15 protein at an initial dilution of 1 μg/mL is also established. After 6 hours, fluorescence intensity is measured using a microplate reader. To validate the blocking efficiency at different concentrations of GDF15, HEK293 SER-Luc2-cRET-GFRAL cells are seeded on plates; KY-NAb-GDF15 antibody at a fixed concentration of 0.1 ng/mL is co-incubated with GDF15 protein starting from an initial dilution of 1 μg/mL for 15 minutes before being added to the pre-seeded plate containing HEK293 SER-Luc2-cRET-GFRAL cells. A negative control group with only GDF15 protein at an initial dilution of 1 μg/mL is also included. After 6 hours, fluorescence intensity is measured using a microplate reader.

### Flow cytometry

The non-specific binding of KY-NAb-GDF15 with peripheral blood monocytes, umbilical vein endothelial cells, red blood cells, and granulocytes was analyzed using flow cytometry. From 08/01/2022 to 08/31/2022, two healthy volunteers were recruited to obtain blood samples. All two healthy volunteers provided written informed consent. Cells were suspended in an antibody dilution solution at a density of 0.2-1x10^5 cells per well and incubated with Human TruStain FcX™. After incubation with the test antibody, washing was performed using an antibody dilution solution. Subsequently, Goat anti-Human IgG Fc conjugated with PE was added for incubation followed by the removal of secondary antibodies in preparation for FACS detection.

### Xenograft mouse model

The mice were housed in ventilated microisolator cages equipped with an automatic watering system providing water, while feed was readily available on a wire feeder. Each mouse was housed individually in an enriched environment, including a loft and nesting material. Environmental conditions were maintained within a temperature range of 20–26°C and a relative humidity range of 40–70%. The mice were subjected to a controlled 12-hour light-dark cycle. Intraperitoneal injection was employed for administration, and anesthesia was not required. Post-procedure, animals showed no signs of pain or distress and were monitored for 5 minutes to confirm the absence of adverse effects. Euthanasia was carried out via CO_2_ asphyxiation in a chamber equipped with a regulated flow meter. Approval for all animal experiments was obtained from the Animal Ethics Committee of the Beijing E-Town Biomedical Park experimental animal center, Beijing, China. All procedures adhered to the Guide for the Care and Use of Laboratory Animals and relevant guidelines and regulations, with reporting following the Animal Research: Reporting of *In Vivo* Experiments (ARRIVE) guidelines for enhanced transparency.

The female severe combined immunodeficiency (SCID) mice, aged 8 weeks, were procured from HFK Bio Co., Ltd. Upon arrival, a one-week acclimatization period was provided in the experimental facility to ensure adaptation to the environment. Tumor implantation commenced when the mice reached approximately 9 weeks of age, exhibiting stable growth and weight gain characteristics. The study duration spanned 6–7 weeks. For tumor implantation, each animal received a subcutaneous injection of HT1080 or LS513 cells suspended in an equal volume of sterile Matrigel matrix gel (Corning, Tewksbury, MA, USA) and phosphate-buffered saline (PBS). Tumor progression was monitored by daily assessment of body weight and measurement of tumor dimensions (length and width) using a caliper. Upon reaching approximately 90% of their initial body weight (around 20–30 days post-implantation), the mice were randomly assigned to treatment groups. These groups received either Human IgG1κ Isotype, Ponsegnomab, or KY-NAb-GDF15 antibody via intraperitoneal injection into the abdominal cavity. The antibody doses were calculated based on 1 mg/kg, 0.3 mg/kg, or 0.1 mg/kg of mouse body weight for the initial administration. Subsequently, after 7–10 days, another intraperitoneal injection of the respective antibodies was administered at doses calculated based on 10 mg/kg, 3 mg/kg, or 1 mg/kg of mouse body weight. Throughout the treatment period, body weight and tumor size were monitored daily to assess response and progression.

In the experiment involving a cachexia model in mice, female C57BL/6 mice aged 8 weeks were allocated into three groups: Group A (5 mice), Group B (10 mice), and Group C (10 mice). Daily measurements of each mouse’s body weight were conducted, and the resultant changes were meticulously recorded. Groups B and C received weekly intraperitoneal injections of cisplatin at a dosage of 4 mg/kg, whereas Group A did not receive any cisplatin injections. Subsequently, when the body weight of mice in Groups A and C dropped to approximately 90% of their initial weight (around 28 days post-initiation), they were intraperitoneally administered with the antibody KY-NAb-GDF15 at a dose of 20 mg/kg, twice weekly. In contrast, mice in Group B received intraperitoneal injections of Human IgG1, κ Isotype Control antibody at a matching dosage of 20 mg/kg, also administered twice weekly.

## Results

### Binding affinity and GDF15-GFRAL signaling blocking function of KY-NAb-GDF15

**Fig 1:**
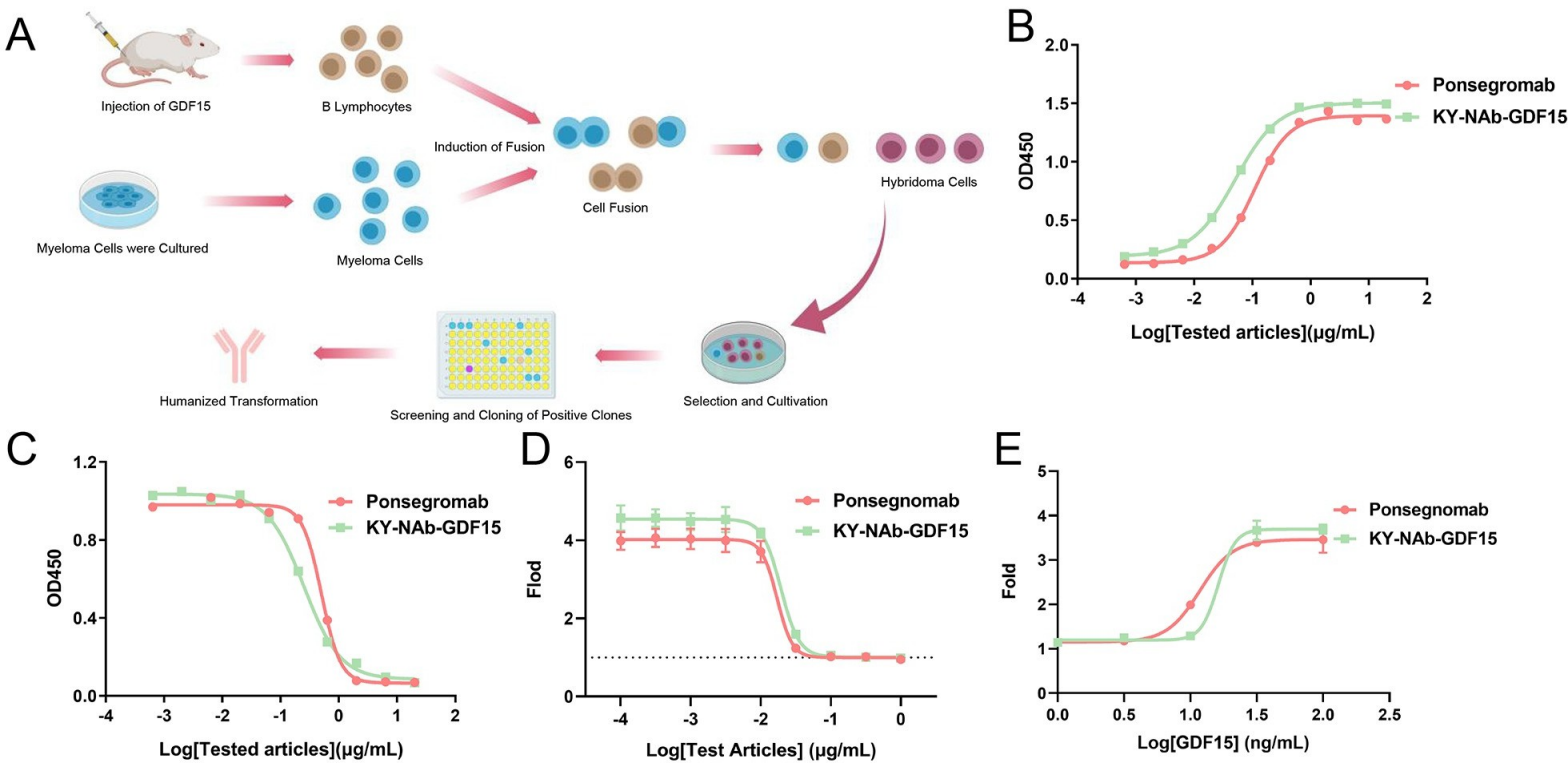
**(A)** Schematic representation of the preparation process for KY-NAb-GDF15, a neutralizing antibody against GDF15. **(B-C)** ELISA assays evaluating the binding and blocking activities of KY-NAb-GDF15. **(B)** Both KY-NAb-GDF15 and Ponsegromab exhibited significant binding activity towards GDF15, with EC50 values of 0.05080 μg/mL and 0.1095 μg/mL, respectively. **(C)** Both KY-NAb-GDF15 and Ponsegromab demonstrated effective blocking activity against the interaction between GDF15 and GFRAL, with IC50 values of 0.2525 μg/mL and 0.5037 μg/mL for KY-NAb-GDF15 and Ponsegromab, respectively. **(D)** KY-NAb-GDF15 inhibits GDF15-stimulated GFRAL signaling in the HEK293 SRE-luc2-cRET-GFRAL reporter system with higher efficiency than Ponsegnomab. **(E)** With 0.1 μg/mL antibody concentration, KY-NAb-GDF15 (EC50 = 16.2 ng/mL) neutralized more GDF15 than Ponsegnomab (EC50 = 11.80 ng/mL).

The discovery process of KY-NAb-GDF15 is depicted in Fig 1A. To assess the antigen-antibody affinity of KY-NAb-GDF15, Bio-Layer Interferometry technology was employed. The results (S1 Fig) showcased a robust binding interaction between KY-NAb-GDF15 and its corresponding antigen, with a dissociation constant (Kd) of 5.26E-11. This affinity is comparable to that of the control antibody Ponsegromab (Kd < 1.0E-12). Furthermore, the efficacy of the anti-GDF15 antibody KY-NAb-GDF15 was scrutinized using ELISA, in conjunction with blocking and cell binding assays. The results obtained from the ELISA assay (Fig 1B) illustrate that KY-NAb-GDF15 specifically targets the GDF15 protein, showcasing a comparatively lower EC50 value than the control antibody Ponsegromab. This suggests that KY-NAb-GDF15 requires a lower concentration to neutralize the same amount of GDF15 compared to Ponsegromab. Furthermore, findings from the blocking assay (Fig 1C) reveal that both KY-NAb-GDF15 and Ponsegromab effectively inhibit the binding between hGDF15-mFC and GFRAL protein. This implies that KY-NAb-GDF15 efficiently impedes downstream reactions triggered by the interaction between GDF15 and GFRAL.

To better simulate the cellular-level neutralizing effect of KY-NAb-GDF15, we established a corresponding reporter cell line, HEK293 SRE-Luc2-cRET-GFRAL (S2 Fig), for GDF15 detection. The results presented in Fig 1D demonstrate a decrease in the binding between GDF15 protein and reporter cells as the concentration of neutralizing antibody increases. This indicates that KY-NAb-GDF15 effectively disrupts the interaction between GDF15 and cell surface GFRAL, consequently inhibiting downstream reactions. This effect is consistent with ELISA blocking experiments and comparable to control antibodies. Additionally, utilizing the reporter cell line depicted in Fig 1E, we conducted GDF15 neutralization experiments. Consistently, with a constant antibody concentration, stronger fluorescence signals were observed as the concentration of GDF15 protein increased within the system. This outcome indicates the effective neutralization of GDF15 by KY-NAb-GDF15, thereby impeding its interaction with cell surface GFRAL and subsequent downstream reactions. However, it’s noteworthy that when the concentration of GDF15 in the system reaches a sufficiently high level, all available KY-NAb-GDF15 becomes utilized, leading to the generation of fluorescence signals. Both ELISA and reporter cell experiments collectively affirm that KY-NAb-GDF15 exhibits exceptional binding specificity and neutralizing efficacy, akin to the control antibody Ponsegromab.

### Specificity of KY-NAb-GDF15

**Fig 2:**
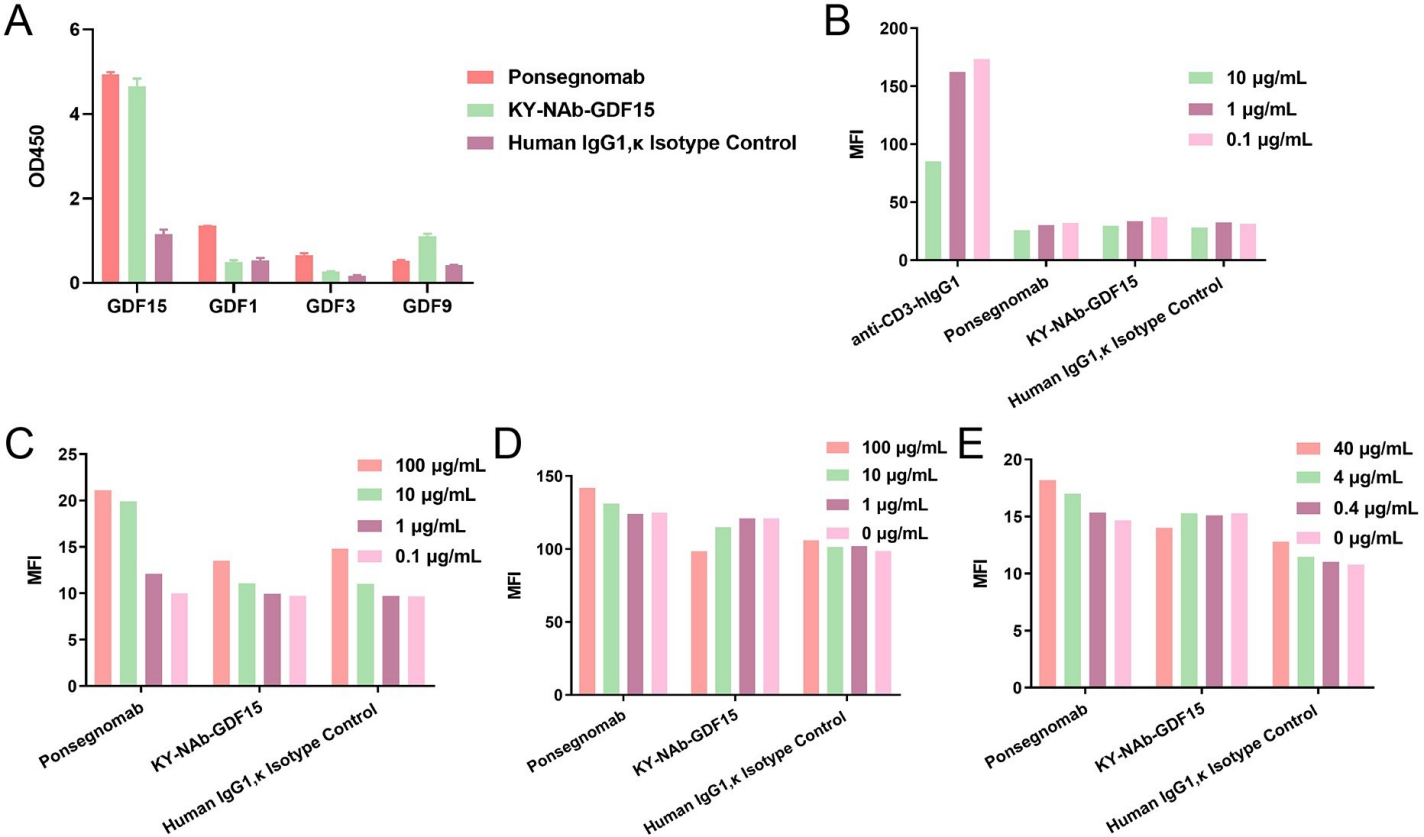
Analysis of the specific binding of KY-NAb-GDF15. **(A)** ELISA assay evaluating the interaction between KY-NAb-GDF15 and GDF family proteins. **(B-E)** Flow cytometry analysis assessing the binding affinity of KY-NAb-GDF15 towards peripheral blood mononuclear cells **(B)**, umbilical vein endothelial cells **(C)**, red blood cells **(D)**, and granulocytes **(E)**.

We conducted an assessment of the binding specificity of KY-NAb-GDF15 with other proteins within the growth differentiation factor family, peripheral blood mononuclear cells, umbilical vein endothelial cells, red blood cells, and granulocytes. Using ELISA, we scrutinized the binding specificity of KY-NAb-GDF15 towards GDF15, GDF1, GDF3, and GDF9. KY-NAb-GDF15 demonstrated significant affinity for its intended target protein GDF15 while exhibiting negligible affinity for other proteins within the same family (Fig 2A). Flow cytometry analysis revealed non-specific binding of KY-NAb-GDF15 to peripheral mononuclear cells, umbilical vein endothelial cells, red blood cells, and granulocytes, as shown in Fig 2B and 2E. In line with Ponsegromab results, the neutralizing antibody KY-NAb-GDF15 did not exhibit significant binding in these cellular environments. These findings underscore that KY-NAb-GDF15 possesses robust binding specificity and a low propensity for off-target interactions in complex *in vivo* settings.

### Stability analysis of KY-NAb-GDF15

We conducted a comprehensive series of stability tests on the KY-NAb-GDF15 antibody under various stress conditions. The results are summarized in Table 1. Notably, no significant changes were observed in SEC-HPLC purity analysis for both treated groups (five freeze-thaw cycles, pH 3.6 treatment, high temperatures for one week and two weeks) and untreated samples. In ELISA binding experiments (S3A Fig), we noted gradual increases in OD450 values with increasing concentrations of the KY-NAb-GDF15 antibody, while there were no notable differences in EC50 values among different treatment types. To account for the complexity of the blood environment, functional activity validation was performed using both PBS and plasma (S3B and S3C Fig). Remarkably, KY-NAb-GDF15 exhibited comparable binding activity to the control antibody Ponsegromab in both PBS and plasma environments.

**Table 1:** Stability assessment of KY-NAb-GDF15 antibody using SEC-HPLC and ELISA techniques following various treatments. The stability testing of KY-NAb-GDF15 was performed under different acceleration conditions, including 5 freeze-thaw cycles, low pH treatment, high-temperature treatment for 1 week and 2 weeks, as well as stability analysis in plasma. Before and after treatment, KY-NAb-GDF15 was analyzed using SEC-HPLC and ELISA to detect changes in integrity and binding function.

Mode of Treatment	Concentration of Antibody (mg/mL)	SEC-HPLC			ELISA Binding Assay
		High Molecular Weight/%	Main PEAK/%	Low Molecular Weight /%	EC50(μg/mL)
**Before treatment**	10.457	5.5272	94.4728	-	0.05878
**Thaw 5 times**	10.568	4.3365	95.6635	-	0.06476
**pH 3.6**	5.004	4.2869	95.7131	-	0.06991
**40°C, 1 week**	10.897	5.3201	94.6799	-	0.06408
**40°C, 2 weeks**	10.784	5.3533	94.6467	-	0.05734
**PBS**	-	-	-	-	0.1272
**Plasma**	-	-	-	-	0.03725

### *In vivo* activity of KY-NAb-GDF15 neutralizing therapy

**Fig 3:**
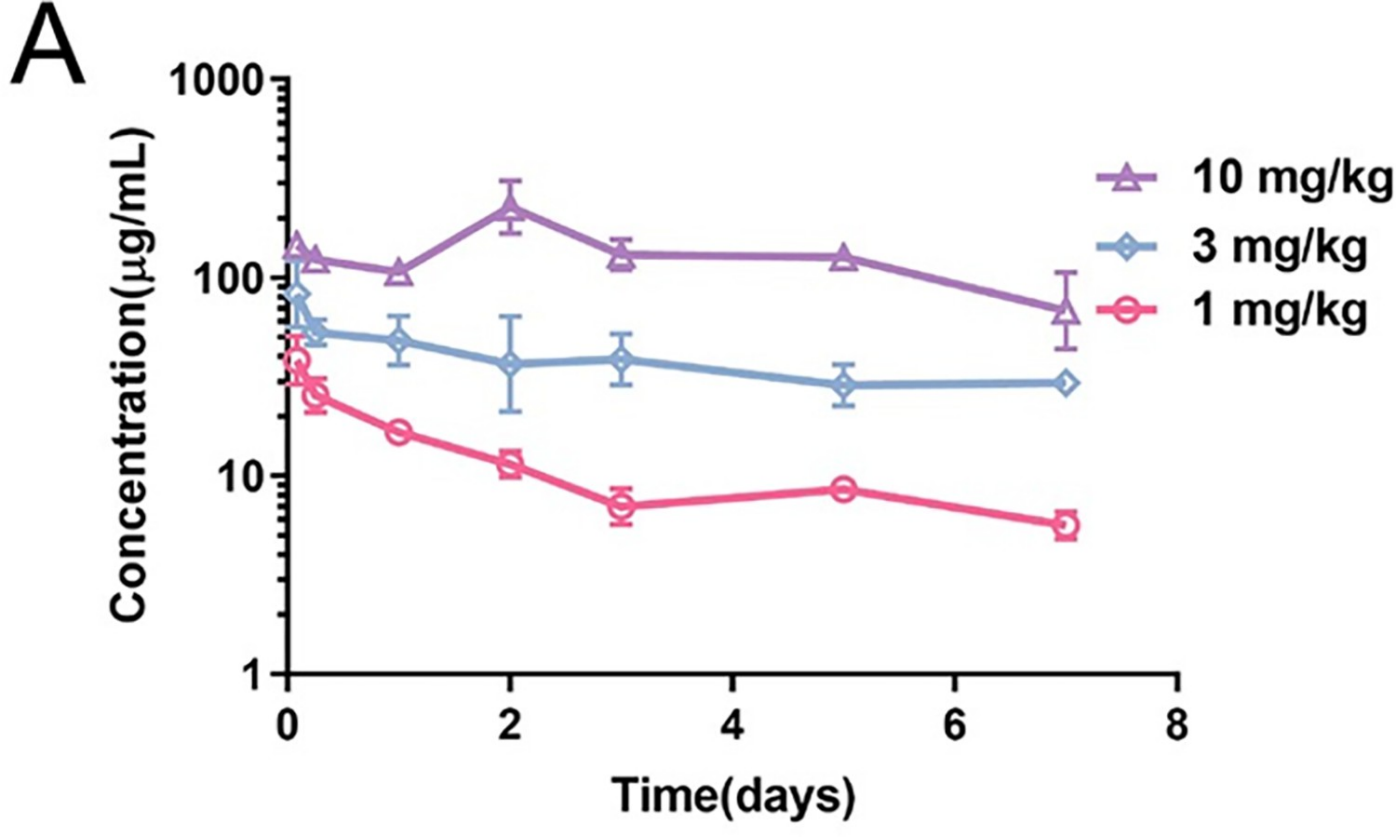
*In vivo* pharmacokinetic assessment of KY-NAb-GDF15. BALB/c mice received intravenous injections of the antibody KY-NAb-GDF15, and serum antibody levels were quantified using ELISA. The half-life (t1/2) of KY-NAb-GDF15 was determined to be approximately 10.14 days at a concentration of 3 mg/kg.

**Fig 4:**
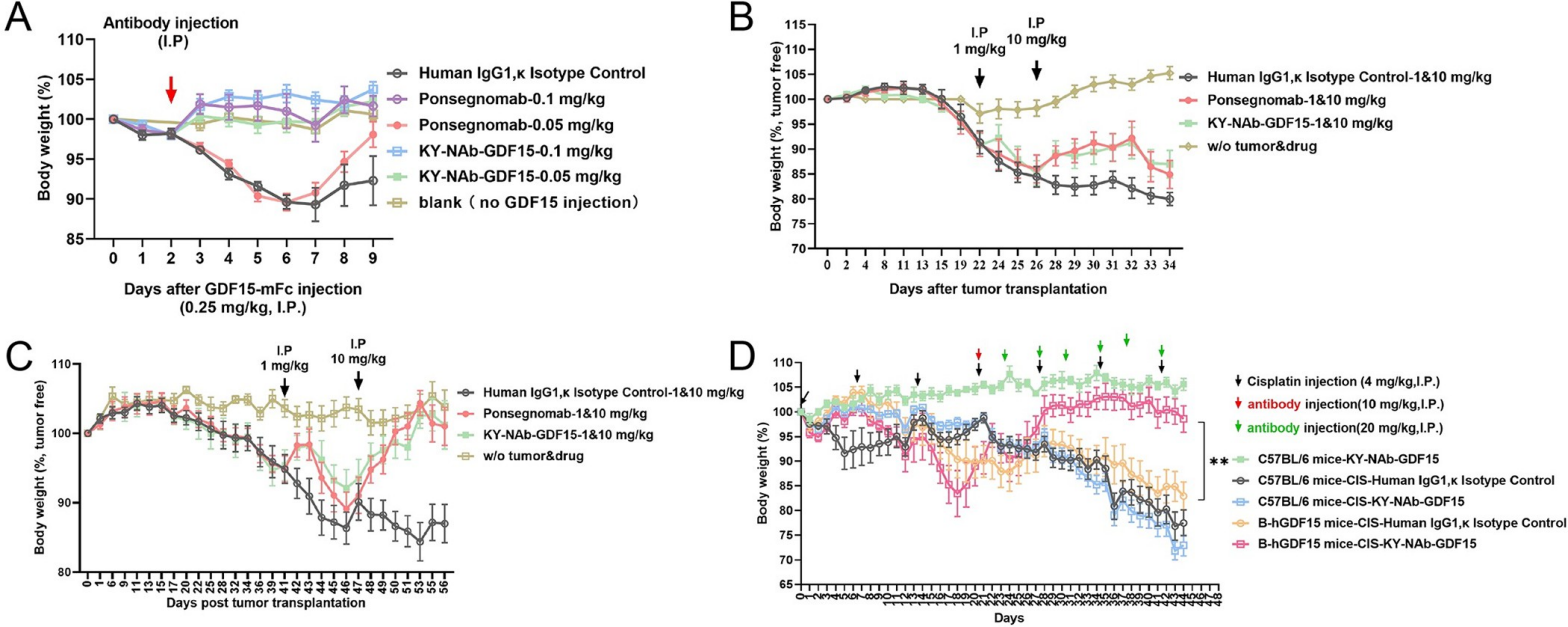
Neutralizing effect of KY-NAb-GDF15 on GDF15-mediated weight loss. **(A)** KY-NAb-GDF15 mitigates GDF15-induced reduction in body weight. BALB/c mice received GDF15-mFc injections on day 0, followed by administration of KY-NAb-GDF15 or Ponsegromab on day 2. The percentage change in body weight was calculated relative to day 0. **(B-C)** KY-NAb-GDF15 attenuates tumor-associated weight loss. Body weight changes in HT1080 xenograft mice **(B)** and LS513 xenograft mice **(C)** following intraperitoneal (IP) injection of KY-NAb-GDF15 or Ponsegromab at doses of 1 mg/kg and 10 mg/kg. The percentage change in body weight was calculated relative to day 0. **(D)** KY-NAb-GDF15 alleviates chemotherapy-induced weight loss. GDF15 humanized mice (B-hGDF15) or WT C57BL/6 mice were treated with cisplatin and KY-NAb-GDF15 or hIgG. The percentage change in body weight was calculated relative to day 0.

In this investigation, we examined the drug metabolism profile of KY-NAb-GDF15 using a BALB/c mouse model. As illustrated in Fig 3, KY-NAb-GDF15 displayed typical pharmacokinetics, with a half-life of 10.14 days following intravenous administration at a dose of 3 mg/kg in mice. To evaluate the neutralizing efficacy of KY-NAb-GDF15, we utilized a BALB/c mouse model injected with GDF15 (Fig 4A). In the absence of GDF15 injection, mouse weight remained consistent throughout the experiment. Introduction of GDF15 resulted in weight loss in mice; however, co-administration with either the neutralizing antibody KY-NAb-GDF15 or Ponsegromab maintained stable mouse weight, indicating effective mitigation against GDF-induced weight loss. Further efficacy assessments of the neutralizing antibody KY-NAb-GDF15 were conducted using tumor animal models. We evaluated its efficacy in human fibrosarcoma mouse models and colorectal cancer mouse models. In both the human fibrosarcoma mouse model (Fig 4B) and colorectal cancer mouse model (Fig 4C), intraperitoneal injection of either KY-NAb-GDF15 antibody or the control antibody Ponsegromab (1 mg/kg and 10 mg/kg) yielded nearly identical results based on the percentage change in body weight per day, while other control groups did not exhibit improvement in the decline in body weight of mice. These findings suggest that the KY-NAb-GDF15 antibody effectively mitigates weight loss induced by tumors. To further validate the efficacy of the neutralizing antibody KY-NAb-GDF15 in alleviating cachexia, experiments were conducted using GDF15 humanized mice and C57BL/6 mouse models. These models received regular injections of the chemotherapy drug cisplatin, and changes in their body weight were monitored. As depicted in Fig 4D, while the control group of C57BL/6 mice without cisplatin injection but treated with KY-NAb-GDF15 maintained stable body weight, all experimental groups injected with cisplatin exhibited a decrease in body weight, indicating that chemotherapy drugs induced cachexia and consequent weight loss in these animals. Upon reaching a certain extent of weight loss, mice were administered antibody drugs. The administration of neutralizing antibody KY-NAb-GDF15 gradually restored their body weight, suggesting its effective targeting of GDF15 and improvement of cisplatin-induced weight loss in mice.

## Discussion

Cachexia is a pathological condition characterized by severe emaciation and systemic weakness, which not only leads to weight loss but also accompanies symptoms such as muscle atrophy, reduced adipose tissue, and malnutrition, severely impacting patients’ quality of life and prognosis [1, 6, 7]. The expression level of GDF15 is closely associated with the occurrence and development of cachexia. Typically, GDF15 levels are significantly elevated in cachectic patients, and its upregulation is thought to be closely related to the physiological and pathological processes associated with cachexia, including muscle degradation and abnormal fat metabolism [31, 32]. GDF15 is considered one of the important biomarkers of cachexia, playing a crucial role in the pathogenesis of cachexia.

As a novel target, research on the development of antibody drugs targeting GDF15 is ongoing. Several pharmaceutical companies have developed corresponding antibody drugs to address this condition. These antibody drugs are still in the research and development stage, including Ponsegromab (Pfizer) [14, 33], CTL-002 (CatalYm) [34], AV-380 (AVEO) [35], etc. In the natural setting, mouse monoclonal antibodies offer distinct advantages, including heightened specificity and simplified production processes, capitalizing on the host animal’s inherent ability to produce highly specific, high-affinity, and fully functional mAbs. Hybridoma screening technology has advantages such as high efficiency, high affinity, diversity, stability, and economy. In our study, we successfully obtained the highly efficient GDF15 neutralizing antibody KY-NAb-GDF15 through its immune preparation. KY-NAb-GDF15 demonstrates strong affinity and effectively blocks downstream signaling mediated by GFRAL induced by GDF15, thereby intervening in subsequent functional changes caused by high concentrations of GDF15. The evaluation of antibody drug quality is heavily dependent on the specificity of their binding to target molecules, as non-specific interactions with proteins or other molecules outside the intended target can lead to severe adverse reactions [36–39]. The complex milieu of human tissues and body fluids presents challenges for antibody drugs [40]. For instance, non-specific binding between antigens and antibodies may result in loss of functionality or undesirable side effects. Most often, such non-specific bindings stem from the interaction between the conserved FC end region of antibodies and cell surface FC receptors (FCRs), expressed on various cells including B cells, monocytes/macrophages, granulocytes, etc. Additionally, off-target binding may occur among structurally similar members within the same protein family. KY-NAb-GDF15 exhibits good binding specificity, effectively identifying and binding to the target, with no significant off-target binding to structurally similar homologous targets. Moreover, there is minimal possibility of off-target interactions in complex *in vivo* environments. Stability is a pivotal parameter in assessing drug quality, significantly influencing the performance, specificity, and affinity of antibody drugs. KY-NAb-GDF15 possesses favorable pharmaceutical properties by demonstrating good thermal stability without aggregation or precipitation under extreme pH conditions, as well as showing negligible impact on its stability from freeze-thaw treatment, laying an important foundation for subsequent drug experiments. The pharmacokinetics of antibody drugs are critical as they significantly influence their duration of action within the body. Optimizing drug metabolism dynamics can prolong the presence of antibody drugs, thereby allowing for sustained therapeutic effects. Importantly, KY-NAb-GDF15 demonstrates superior metabolic stability and therapeutic efficacy in *in vivo* experiments. It effectively alleviates tumor-induced weight loss and similarly alleviates cachexia induced by chemotherapy drugs. Currently, antibody drugs targeting GDF15 on the market are in the development stage. Disclosed drugs include Ponsegromab (Pfizer), CTL-002 (CatalYm), and AV-380 (AVEO), which all function by neutralizing excessive GDF15 to block its subsequent functions. In our study, KY-NAb-GDF15 was compared with Ponsegromab, showing comparable or greater advantages in various aspects, indicating the potential of the newly developed GDF15 neutralizing antibody as a drug.

Although cancer cachexia is associated with GDF15 overexpression, GDF15 itself holds biological significance [10, 41]. GDF15 has been demonstrated to regulate immune responses and exhibit neuroprotective effects. It is relevant to cardiovascular health and associated with conditions such as heart failure and atherosclerosis [42, 43]. Consequently, determining the optimal dosage for GDF15 neutralizing antibodies becomes a critical research focus. Evaluating the target specificity of biologic therapies, including KY-NAb-GDF15, is essential to minimize off-target effects during subsequent drug development stages.

Due to the close correlation between the expression levels of GDF15 in various diseases and the severity and prognosis of the diseases, researchers are also exploring the potential application of GDF15 as a biomarker for disease diagnosis and prognosis assessment [27, 44–46]. For example, using the ELISA method to detect the content of GDF15 in samples to determine the likelihood of disease occurrence. This accompanying diagnostic method can be used for the detection of the disease biomarker GDF15 in human physical examinations, serving as a preliminary screening for cancer or inflammation. In the development of GDF15 ELISA detection kits, antibodies with high specificity and multiple binding epitopes are key factors in the detection method. The favorable properties of KY-NAb-GDF15 also provide possibilities for its application in the development of detection kits.

In summary, the performance of the neutralizing antibody KY-NAb-GDF15 is remarkable, showing great potential in the treatment of cancer cachexia and in GDF15-related detection fields.

## Supporting information

S1 Fig Affinity detection results of antibodies and GDF15. Ponsegromab **(A)** and KY-NAb-GDF15 **(B)** were selected based on discrimination using five concentration curves: 200 nM, 100 nM, 50 nM, 6.25 nM, and 3.12 nM. Additionally, affinity detection results of Ponsegromab, KY-NAb-GDF15, and Human GDF15-His protein **(C)** are provided.
(PDF)

S2 Fig Construction of the HEK293 SRE-luc2-cRET-GFRAL reporter system. **(A)** Western Blot analysis was conducted to determine the expression level of GFRAL-RET in the HEK293 SRE-luc2-cRET-GFRAL reporter system. **(B)** Interaction of RET with GFRAL on the cell surface driven by GDF15 is demonstrated.
(PDF)

S3 Fig Antibody KY-NAb-GDF15 stability testing for binding activity. The ELISA method was employed to assess the binding activity of KY-NAb-GDF15 under various stress conditions: repeated freeze-thaw cycles, low pH, high-temperature treatment **(A)**, PBS **(B)**, and plasma **(C)**.
(PDF)

S4 Fig Tumor volume of HT1080 xenograft mice **(A)** and LS513 xenograft mice **(B)** with intraperitoneal (IP) Injection of KY-NAb-GDF15 or Ponsegromab at doses of 1 mg/kg and 10 mg/kg.
(PDF)

S1 Raw images Western Blot raw image for S2A Fig. (PDF)
