# Supplementary material for: Neutralizing antibody against GDF15 for treatment of cancer-associated cachexia
Source: PLoS One. 2024 Aug 22;19(8):e0309394. doi: 10.1371/journal.pone.0309394 (PMC11341059; doi:10.1371/journal.pone.0309394)
Supplement: S1 Fig — Ponsegromab (A) and KY-NAb-GDF15 (B) were selected based on discrimination using five concentration curves: 200 nM, 100 nM, 50 nM, 6.25 nM, and 3.12 nM. Additionally, affinity detection results of Ponsegromab, KY-NAb-GDF15, and Human GDF15-His protein (C) are provided. (PDF) [file pone.0309394.s001.pdf]

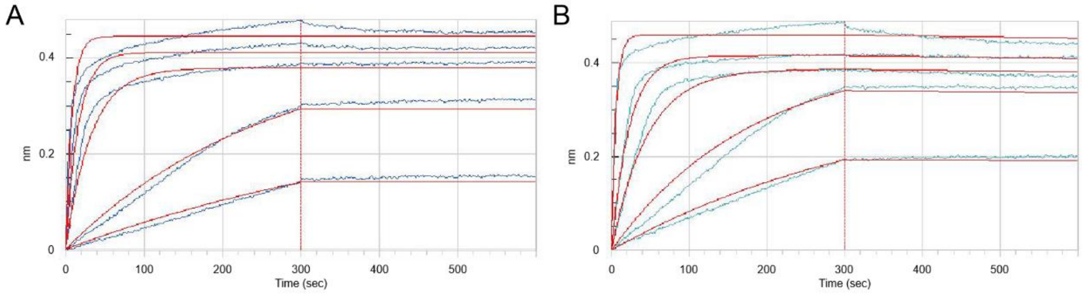

C

Protein name	KD/M	Kon(1/Ms)	Kdis(1/s)
Ponsegnomab	<1.0E-12	6.21E+05	<1.0E-07
KY-NAb-GDF15	5.26E-11	8.74E+05	4.60E-05

S1 Fig. Affinity Detection Results of Antibodies and GDF15. Ponsegromab **(A)** and KY-NAb-GDF15 **(B)** were chosen based on their effective discrimination using five concentration curves at 200 nM, 100 nM, 50 nM, 6.25 nM, and 3.12 nM. The affinity detection results for Ponsegromab, KY-NAb-GDF15, and Human GDF15-His protein **(C)**.
