# Supplementary material for: Neutralizing antibody against GDF15 for treatment of cancer-associated cachexia
Source: PLoS One. 2024 Aug 22;19(8):e0309394. doi: 10.1371/journal.pone.0309394 (PMC11341059; doi:10.1371/journal.pone.0309394)
Supplement: S2 Fig — (A) Western Blot analysis was conducted to determine the expression level of GFRAL-RET in the HEK293 SRE-luc2-cRET-GFRAL reporter system. (B) Interaction of RET with GFRAL on the cell surface driven by GDF15 is demonstrated. (PDF) [file pone.0309394.s002.pdf]

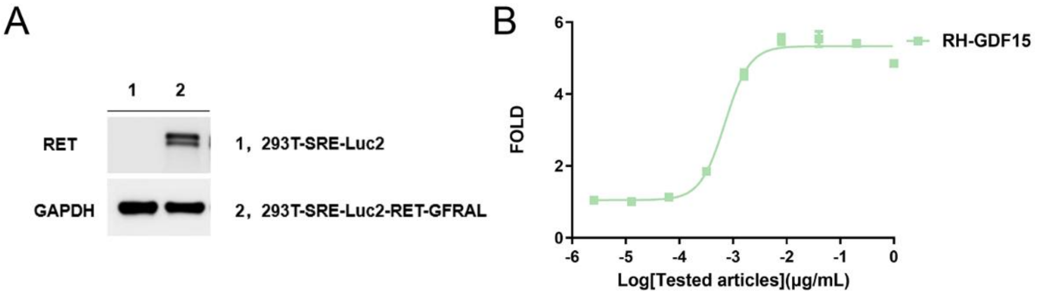

S2 Fig. Construction of the HEK293 SRE-luc2-cRET-GFRAL reporter system. (A) Western Blot analysis was performed to determine the expression level of GFRAL-RET in the HEK293 SRE-luc2-cRET-GFRAL reporter system. **(B)** GDF15-driven interaction of RET with GFRAL on the cell surface.
