# Supplementary material for: Neutralizing antibody against GDF15 for treatment of cancer-associated cachexia
Source: PLoS One. 2024 Aug 22;19(8):e0309394. doi: 10.1371/journal.pone.0309394 (PMC11341059; doi:10.1371/journal.pone.0309394)
Supplement: S3 Fig — The ELISA method was employed to assess the binding activity of KY-NAb-GDF15 under various stress conditions: repeated freeze-thaw cycles, low pH, high-temperature treatment (A), PBS (B), and plasma (C). (PDF) [file pone.0309394.s003.pdf]

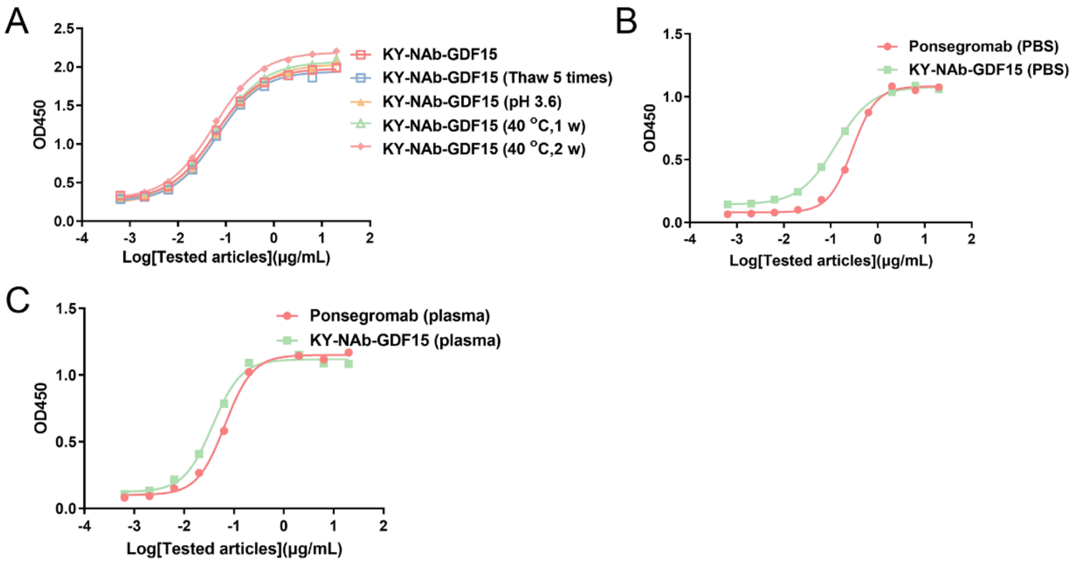

S3 Fig. Antibody KY-NAb-GDF15 stability testing for binding activity. ELISA method was used to assess the binding activity of KY-NAb-GDF15 under different stress conditions: repeated freeze-thaw cycles, low pH, high temperature treatment **(A)**, PBS **(B)**, and plasma **(C)**.
