# Supplementary material for: Neutralizing antibody against GDF15 for treatment of cancer-associated cachexia
Source: PLoS One. 2024 Aug 22;19(8):e0309394. doi: 10.1371/journal.pone.0309394 (PMC11341059; doi:10.1371/journal.pone.0309394)
Supplement: S4 Fig — Tumor volume of HT1080 xenograft mice (A) and LS513 xenograft mice (B) with intraperitoneal (IP) Injection of KY-NAb-GDF15 or Ponsegromab at doses of 1 mg/kg and 10 mg/kg. (PDF) [file pone.0309394.s004.pdf]

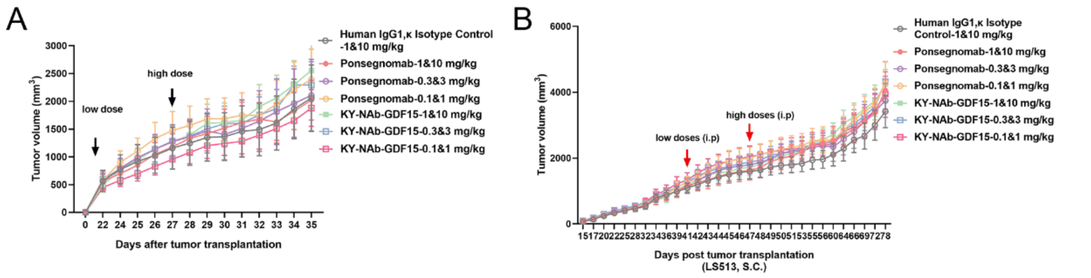

S4 Fig. Tumor volume of HT1080 xenograft mice **(A)** and LS513 xenograft mice **(B)** with intraperitoneal (IP) Injection of KY-NAb-GDF15 or Ponsegromab at 1 mg/kg, 3 mg/kg and 10 mg/kg.
