# Supplementary figures and images for: Neutralizing antibody against GDF15 for treatment of cancer-associated cachexia
Source: PLoS One. 2024 Aug 22;19(8):e0309394. doi: 10.1371/journal.pone.0309394 (PMC11341059; doi:10.1371/journal.pone.0309394)

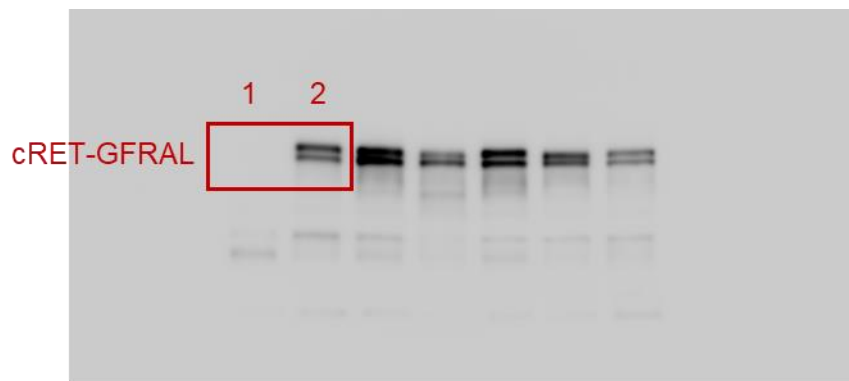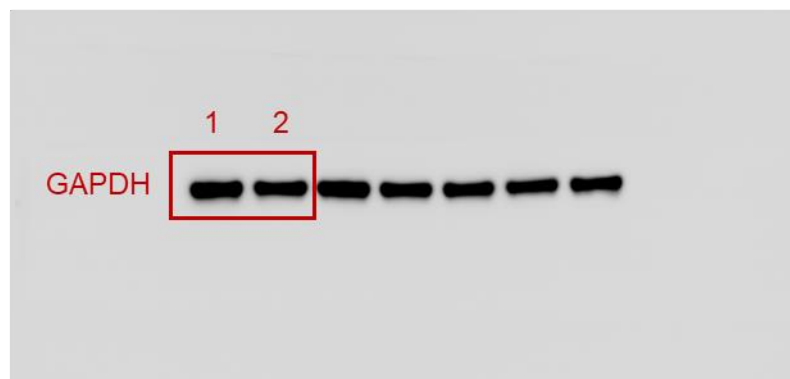

1. HEK293 SRE-luc2
2. HEK293 SRE-luc2-cRET-GFRAL

Supplement: S1 Raw images — (PDF) [file pone.0309394.s005.pdf]
